# Characterization of the complete mitochondrial genome sequence of *Coilia brachygnathus* (Clupeiformes, Engraulidae) from Huai River and its phylogenetic position

**DOI:** 10.1080/23802359.2022.2097487

**Published:** 2022-07-12

**Authors:** Yuan Tian, Tiezhu Yang, Shijie Yang, Leping Wang, Liangjie Zhao, Wenhao Sun

**Affiliations:** a School of Fisheries, Xinyang Agriculture and Forestry University, Xinyang, China; b Fishery Biological Engineering Technology Research Center of Henan Province, Xinyang, China; c Xinyang Nanwan Reservoir Fishery Development Co. Ltd., Xinyang, China; d Bureau of Agriculture and Rural Affairs of the Zhumadian City, Zhumadian, China

**Keywords:** *Coilia brachygnathus*, mitochondrial genome, Huai River, phylogenetic analysis

## Abstract

The complete mitogenome sequence of *Coilia brachygnathus* (Kreyenberg & Pappenheim, 1908) from Wabu Lake in Huai River Basin was annotated and characterized in this study. This mitochondrial genome is a circular DNA molecule of 16.896 bp in size with 57.52% AT content, including 13 protein-coding genes (PCGs), two ribosomal RNA genes (rRNAs), 22 transfer RNA genes (tRNAs), and an AT-rich region (control region) as other bony fishes. There are a total of 10 overlap locations and 15 intergenic spacer regions throughout the mitogenome of *C. brachygnathus*. All PCGs employed a standard ATG as a start codon, except cytochrome c oxidase 1 (cox1) with GTG. In addition, TAA or TAG was identified as the typical stop codon. A phylogenetic tree reconstructed with the maximum likelihood method depicted a clone relationship with eight species of genus *Coilia* and our previous study based on the amino acid sequences of 13 mitochondrial PCGs. The complete mitochondrial genome is a valuable resource in classifying and conserving *C. brachygnathus*.

*Coilia brachygnathus* (Clupeiformes: Engraulidae: Coilinae), known as the only freshwater fish in the genus *Coilia*, is a kind of small fish growing, developing, and reproducing in Yangtze River and its affiliated water bodies (Whitehead 1985). The Huai River which flows into the Yangtze River at the lower reaches of Hongze Lake is known as one of the affiliated water bodies. It was shown by previous investigations that these species were abundant in the Huai River. This species is an important food source for local populations, as well as a bait for large carnivorous fish, *C. brachygnathus* plays an important role in the food chain in the Huai River (Wang et al. 2020). It is acknowledged that under the influence of various factors, such as overfishing, environmental pollution, and habitat destruction, the wild resources of *C. brachygnathus* were under great threat. Till now, although the multiple genomes of *C. brachygnathus* from the Huai River have been published, the populations they sampled belonged to the migratory ones in the mainstream of the Huai River rather than the land-locked population, while the populations living in the Wabu Lake were typical land-locked (Hu et al. 2019). There is no denying the fact that our achievement will facilitate further studies on *Coilia* fishes, including the phylogenetic relationship between the species and population structure of *C. brachygnathus* from the Huai River.

The complete mitochondrial genome of *C. brachygnathus* was sequenced and characterized in this study. The samples of *C. brachygnathus* were collected from the Wabu Lake (E116.9076 and N32.3861) in Huainan City, Anhui Province, China, and stored in the Aquatic Museum of Xinyang Agriculture and Forestry University (https://www.xyafu.edu.cn/, Liangjie Zhao and a850924t@163.com) under the voucher number XYAFU-Mu-1803226. Before further processing, the samples were placed in 100% ethanol in the process of collection and were stored at ambient temperature (−80 °C) until the extraction of DNA. The extraction of total DNA from the partial body tissue was carried out employing the TIANamp Genomic DNA Kit (Tiangen Biotech, Beijing, China). With the quality of the separated DNA detected by 1.5% electrophoresis, the DNA was stored at ambient temperature (−20 °C) until the complete mitogenomes were amplified by PCR. Long and accurate PCR (LA PCR) was performed to amplify the complete mitochondrial genome sequence using a set of specific primers. Sequencing was conducted using the primer walking method on an ABI 3730XL DNA Analyzer (Applied Biosystems Inc., Foster City, CA, USA).

With a length of 16.896 bp, the complete mitochondrial genome of *C. brachygnathus* has been deposited in GenBank with accession No. ON209111. It is composed of 13 protein-coding genes, 22 tRNA genes, and two rRNA genes. The encoding genes of mitogenome were located on H-strand except for nad6 and eight tRNA genes, which were transcribed from L-strand. With only cox1 starting with GTG, the ATG and GTG are used as the start codon. Except for cox2, nad4, and Cytb with an incomplete stop codon ‘T–,’ the remaining protein-coding genes stop with TAG or TAA. The tRNA genes were interspersed among the mitochondrial genome, with the sizes ranging from 66 to 75 bp, and it was found that twenty-two tRNA genes were folded into a typical cloverleaf secondary structure (Donath et al. 2019), which was similar to other *Coilia* mitochondrial genomes (Hu et al. 2019; Yang et al. 2019). It was estimated that the overall basic composition of the heavy strand in *C. brachygnathus* was 31.21% for A, 26.30% for T, 15.47% for G, and 27.01% for C, with an AT content of 57.52%, respectively. As a result, it indicated that there was an obvious antiguanine bias. The 13 PCGs have a size of 11.424 bp in total, accounting for 67.61% of the whole mitogenome.

We selected eight complete mitogenome data of *Coilia* obtained from the GenBank database. In addition, *Thryssa kammalensis* (Bleeker, 1849) (KU761588) was deemed as an outgroup. Apart from that, PartitionFinder 2.1.1 was employed to select the optimal evolutionary models for phylogenetic analysis (Lanfear et al. 2017). The preparation for the amino acid sequence of the 13 genes for all the selected species was conducted in the following specific order: nad1, nad2, cox1, cox2, atp8, atp6, cox3, nad3, nad4L, nad4, nad5, nad6, and cob. Phylogenetic analysis was carried out using the IQ-TREE v1.6.10 (Minh et al. 2020). It was shown by the results (Figure 1) that the sample from Wabu Lake should be classified as *C. brachygnathus*, and it is a member of the genus *Coilia*.

**Figure 1.**
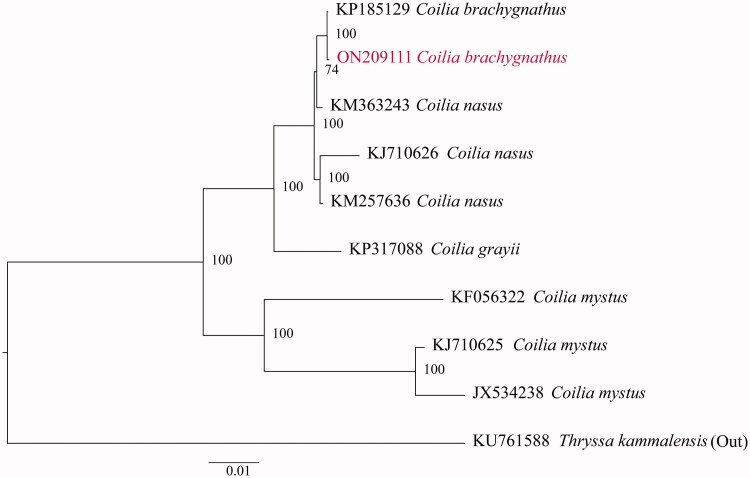
Phylogenetic tree generated using the IQtree method from the amino acid composition of the complete mitochondrial genomes. The red font represents the *C. brachygnathus* from Wabu Lake.

## Data Availability

The genome sequence data that support the findings of this study are openly available in GenBank of NCBI at https://www.ncbi.nlm.nih.gov/ under the accession no. ON209111.
